# Amorphous Carbon Generation as a Photocatalytic Reaction on DNA-Assembled Gold and Silver Nanostructures

**DOI:** 10.3390/molecules24122324

**Published:** 2019-06-24

**Authors:** Christian Heck, Yuya Kanehira, Janina Kneipp, Ilko Bald

**Affiliations:** 1 Institute of Chemistry, University of Potsdam, Karl-Liebknecht-Str. 24–25, 14476 Potsdam, Germany; check@tauex.tau.ac.il (C.H.); yuya.kanehira@uni-potsdam.de (Y.K.); 2 BAM Federal Institute for Materials Research and Testing, Richard-Willstätter-Str. 11, 12489 Berlin, Germany; janina.kneipp@chemie.hu-berlin.de; 3 Department of Chemistry & SALSA, Humboldt Universität zu Berlin, Brook-Taylor-Str. 2, 12489 Berlin, Germany

**Keywords:** amorphous carbon, DNA origami, SERS, nanoparticle dimers, nanolenses

## Abstract

Background signals from in situ-formed amorphous carbon, despite not being fully understood, are known to be a common issue in few-molecule surface-enhanced Raman scattering (SERS). Here, discrete gold and silver nanoparticle aggregates assembled by DNA origami were used to study the conditions for the formation of amorphous carbon during SERS measurements. Gold and silver dimers were exposed to laser light of varied power densities and wavelengths. Amorphous carbon prevalently formed on silver aggregates and at high power densities. Time-resolved measurements enabled us to follow the formation of amorphous carbon. Silver nanolenses consisting of three differently-sized silver nanoparticles were used to follow the generation of amorphous carbon at the single-nanostructure level. This allowed observation of the many sharp peaks that constitute the broad amorphous carbon signal found in ensemble measurements. In conclusion, we highlight strategies to prevent amorphous carbon formation, especially for DNA-assembled SERS substrates.

## 1. Introduction

Since the early studies on surface-enhanced Raman scattering (SERS), amorphous carbon has been known as a potential contaminant that generates intense, broad background signals [1,2]. Amorphous carbon signals have been reported for SERS on gold and silver nanostructures [3,4] and were described to originate from initial carbon contamination on the metal surface [5], or to be created in situ by photodegradation under the highly-enhanced fields [1,2,6]. Amorphous carbon is made up from various carbon species, whose broad ensemble spectrum resembles that of graphite [7,8]. Especially in single-molecule SERS and tip-enhanced Raman spectroscopy, amorphous carbon contaminations proved problematic [9]. The intense, fluctuating SERS signals from individual carbon species are easily mistaken for spectral blinking of single analyte molecules [4]. They show strong signals due to resonant excitation [1,10]. Although amorphous carbon is frequently observed in SERS measurements at low molecular concentrations, so far there is no complete understanding of the circumstances of its formation. DNA origami can assemble plasmonic nanoparticles into aggregates with defined geometry and stoichiometry [11,12]. As the shape and material of a plasmonic nanostructure determines the amplitude and resonance wavelength of its localized surface plasmon resonances (LSPRs), DNA origami-based plasmonic nanostructures represent versatile models for fundamental studies on plasmonic nanosystems. DNA origami-based SERS is an emerging field and has potential to offer dedicated platforms for reproducible single-molecule spectroscopy and detection [13,14,15,16,17]. The present study discusses the formation of amorphous carbon on DNA origami-assembled plasmonic nanostructures for several different experimental conditions.

## 2. Results

**Figure 1:**
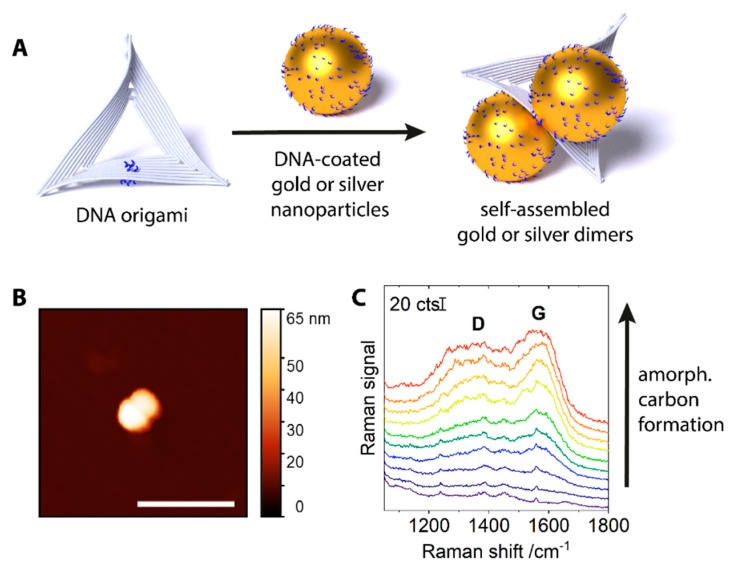
(**A**) Assembly scheme for 60 nm gold and silver dimers. (**B**) Atomic force microscopy (AFM) image of a gold dimer. Scale bar: 300 nm. (**C**) Average SERS signals of consecutive scans over an area of 170 × 190 μm^2^ with 60 nm silver dimers, demonstrating the gradual increase of the amorphous carbon signal (bottom to top, D and G band annotated, 532 nm irradiation, 450 kW cm^−2^, 0.2 s integration per spot). Spectra are offset for clarity.

DNA-coated gold or silver nanoparticles of 60 nm diameter were assembled into dimers by triangular DNA origami scaffolds (Figure 1A). On each face, the DNA origami scaffolds carried a group of four single-stranded DNA extensions (A_24_ on 5′-end, blue in Figure 1A) that would bind nanoparticles coated with DNA of a complementary sequence (T_13_-SH, also blue). An atomic force microscopy (AFM) image of an assembled dimer is shown in Figure 1B. The interparticle distance in the dimers is determined by the particle coating and the intermediate DNA origami layer and is estimated to be around 3 nm [13]. The nanostructures were deposited on solid supports (silicon wafers, p-doped with boron, 100 orientation). Raman scans over large areas (170 × 190 μm^2^) were conducted, with the intention to average the signal from as many plasmonic nanostructures as possible and to achieve a representative and reproducible overview, despite the typically inhomogeneous distribution of nanostructures and hot spot intensities over the surface. Judging from AFM measurements, several thousand plasmonic nanostructures (monomers and dimers) were present in a scanned area. (See Figure S1 in the SI.) Time series were created by conducting several consecutive scans over the same area on the substrate. When averaging the spectra from a respective scan and examining the evolution of this average signal, we observed the appearance and gradual increase of amorphous carbon-characteristic signals. Figure 1C gives an example of such data for silver dimers under 532 nm irradiation (450 kW cm^−2^, 0.2 s integration per spot). While the first scan (bottom) does not contain any contribution from amorphous carbon, in the following scans, a gradual increase of a broad background can be observed. It has the typical ‘cathedral’ shape of the spectrum of amorphous carbon, featuring the G-band at ~1580 cm^−1^, and the smaller, broader D-band at ~1350 cm^−1^ [18]. This demonstrates that amorphous carbon is formed in situ, and is not some contamination preexisting on the metal surface [5].

**Figure 2:**
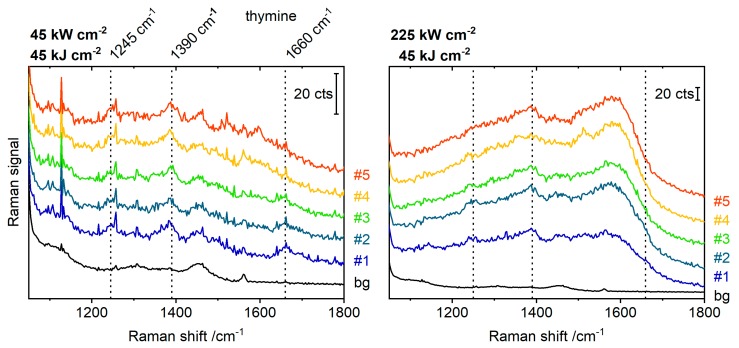
Average SERS signals of five consecutive scans over 60 nm silver dimers with 532 nm excitation. The series were carried out under different irradiation power densities, but the integration time (left: 1 s, right: 0.2 s) was adjusted so that the product of power density and integration time (i.e., the irradiation energy density) would be kept constant at 45 kJ cm^−2^. bg—background signal from a blank silicon wafer. Characteristic thymine vibrations are marked by dotted lines. Spectra are offset for clarity.

In order to examine the relation between amorphous carbon generation and irradiation power density, Raman spectra were measured with different irradiation power densities in raster scans across the sample. For the measurements with higher power densities, the integration time per spot was reduced, so that the product of power density and integration time (that is, the irradiation energy density) would be constant. When an area with silver dimers was exposed to several consecutive Raman scans with 532 nm excitation at low power density (45 kW cm^−2^, 1 s integration, Figure 2), a shallow background signal, characteristic of amorphous carbon, started to appear in the average signal of the fourth scan. This signal is relatively weak and only becomes obvious when the subsequent spectra are overlaid without offset (Figure S2 of the SI). The peaks marked by dotted lines are attributed to thymine, the nucleobase present in the DNA coating of the nanoparticles [19,20]. The measurement was repeated with a fivefold higher laser power density (225 kW cm^−2^) at a different position on the substrate. The integration time per measurement spot was reduced to 0.2 s. Under this increased irradiation power density, already in the first scan, strong bands of carbon are observed which increase in intensity in the average spectrum of the second scan and then stay constant. The identification of thymine, as of any other potential analyte molecule, is impeded by the strong carbon background. Such a favored amorphous carbon formation under high irradiation power densities was also observed for silver dimers under 488 nm irradiation and for gold dimers under 785 nm irradiation. The fact that the higher irradiation power densities are not compensated by the shorter integration times indicates that the formation of amorphous carbon scales non-linearly with the irradiation power. Such a behavior can be attributed to both, thermal excitation and hot electrons originating from the plasmonic nanostructures, which probably act synergistically [21,22]. Hot electrons could populate unoccupied orbitals in the organic adsorbate, transforming it into a transient negative ion, which then could convert to a neutral molecule in an excited vibrational state until it eventually overcomes a reaction barrier. As another possibility, the transient negative ion could dissociate into a negatively-charged fragment and one or more neutral fragments [23]. Our results indicate that decreasing the irradiation power density is more efficient in reducing amorphous carbon formation than shortening of integration times.

For silver dimers under 488 nm and 532 nm illumination, amorphous carbon formed only after several scans, or at high irradiation power densities. In contrast, under 785 nm illumination, even at low power and short integration time, (17 kW cm^−2^, 0.4 s) amorphous carbon signals were visible from the first scan onwards. Interestingly, the choice of substrate on which the structures were deposited played a major role. In a control experiment, where silver dimers were deposited on calcium fluoride instead of silicon and irradiated with the 785 nm laser, no amorphous carbon was formed, even at high laser power densities (520 kW cm^−2^, 1 s, 4 scans). This influence of the silicon substrate was only observed under 785 nm illumination, not with the illumination at 532 nm. Gold dimers, when irradiated with 785 nm under the same condition, did not yield amorphous carbon when deposited on the silicon wafer, even after prolonged exposure (17 kW cm^−2^, 8 s integration time, 2 scans, Figure S3 in the SI). From this, we infer that the strong generation of amorphous carbon under 785 nm illumination cannot be attributed to an effect of the silicon substrate alone. It is possible that due to an interaction between the silver dimers and the silicon substrate at this wavelength, transfer of hot charge carriers to the semiconductor surface can occur [24], leading to a higher efficiency of the photocatalytic process due to a longer lifetime of the hot carriers within the semiconductor.

**Figure 3:**
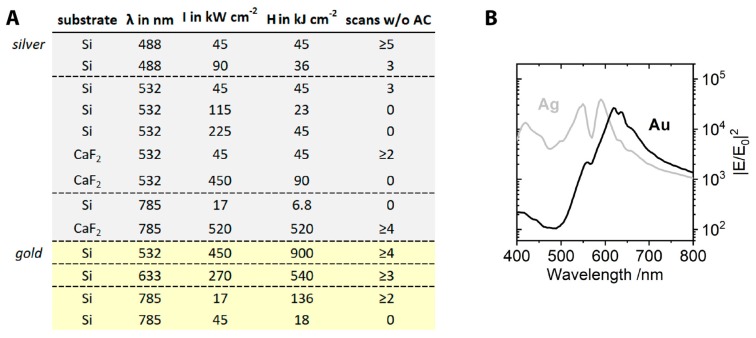
(**A**) Measurement conditions for silver and gold dimers and number of scans before any amorphous carbon (AC) background was observed. λ—irradiation wavelength, I—irradiation power density, H—irradiation energy density. (**B**) Maximal electromagnetic field intensity enhancement of 60 nm gold and silver dimers with a gap of 3 nm, determined by FDTD simulations including the DNA coating and a silicon surface with a 2 nm silicon oxide layer.

In general, gold dimers showed a lower propensity to enhance the formation of amorphous carbon than silver dimers. From the used excitation wavelengths of 532 nm, 633 nm and 785 nm, amorphous carbon was only observed at 785 nm, and only at high power densities (≥43 kW cm^−2^, 0.4 s integration). Figure 3A summarizes some of the conditions tested. Apart from the measurement conditions, it gives the number of scans before any amorphous carbon was observed. A plot of wavelength-dependent field intensity enhancement, simulated for 60 nm gold and silver dimers (gap size 3 nm) is shown in Figure 3B. At wavelengths above 607 nm, the enhancement provided by the gold dimers is higher than in silver dimers. Nevertheless, we observed a preferred amorphous carbon formation on silver dimers at all excitation wavelengths. This indicates that other factors than the heat and intense fields related to the excitation of LSPRs may contribute to the amorphous carbon formation [25,26]. The higher affinity of silver to oxygen and anions is likely to play an important role here as well [27,28]. Szczerbiński et al. recently demonstrated that the photocatalytic degradation of thiol-anchored nanoparticle coatings is influenced by their desorption behavior [29], ascribing to the hot carriers an important role in the desorption of adsorbate molecules and their transformation into reactive, partly radical species. The reaction of these radical species then is hypothesized to create the amorphous carbon layer [29]. In our experiments, the thiol-bond that attaches the DNA coating to the particle surface is considerably weaker on silver than on gold [30]. We conclude for the data shown here that a different desorption behavior could be an important factor in the preferred amorphous carbon generation on silver compared to gold.

**Figure 4:**
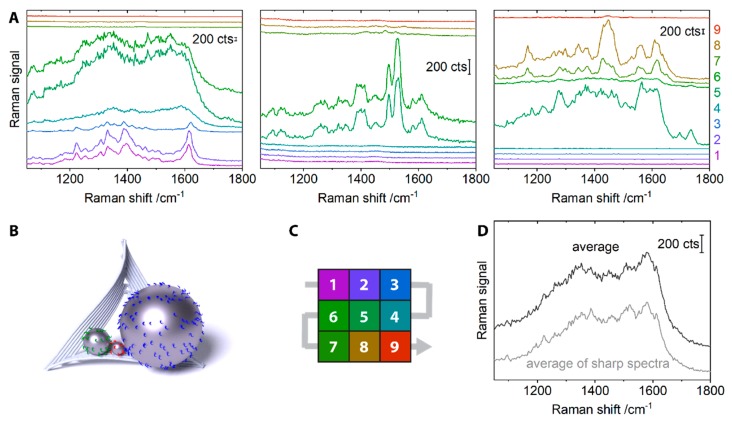
(**A**) Each panel shows the evolvement of the SERS spectrum of a single silver nanolens over time (532 nm illumination, 4 s, left and right: 685 kW cm^−2^, middle: 100 kW cm^−2^). The nine spectra in each panel originate from the nine different positions during the Raman mapping around the single nanolens. Since the laser spot is larger than the raster size of the scan, each nanolens is sampled several times, thereby enabling us to create a time series from the consecutively sampled positions. (**B**) Schematic representation of a DNA origami-assembled silver nanolens. (**C**) Scan path around a single silver nanolens (located at position 5). Numbers and colors correspond to the ones in the SERS spectra under (A). (**D**) Averaged SERS spectra from the twelve single silver nanolenses irradiated at 685 kW cm^−2^. For the grey spectrum, only those spectra with distinct peaks were included. The broad double peak shape that is characteristic for amorphous carbon persists. All spectra are offset for clarity.

The nature of the amorphous carbon spectra was further investigated with a different type of plasmonic aggregate, consisting of three differently-sized silver nanoparticles (60 nm, 20 nm and 10 nm, termed nanolenses [31]. The structures were assembled as described before [16] and deposited on silicon substrates. SERS spectra from individual plasmonic nanostructures were recorded, as confirmed by correlated AFM-Raman mapping (532 nm illumination, 4 s integration per spot). High power densities (100 kW cm^−2^ / 685 kW cm^−2^) ensured the formation of amorphous carbon in a single scan. Spectra from sixteen single silver nanolenses were collected this way. Since the laser spot diameter exceeded the step size used for the map (0.72 µm spot diameter vs. 0.5 µm step size), the nanostructures were sampled several times during the Raman mapping. The scan path of the Raman mapping around a single nanolens is illustrated in Figure 4C, with the single silver nanolens located at position five. The nine sampling positions during the scan around a particular nanolens allow us to follow how its SERS spectrum evolved over time. Data sets for three single silver nanolenses are shown in the three panels of Figure 4A and an illustration of an assembled nanostructure is given in Figure 4B. The left panel in Figure 4A shows the transformation of a spectrum with distinct peaks into a broad band attributed to the formed carbon. As the scan path during the Raman mapping passes the single silver nanolens for the first time (Figure 4A left, spectra 1–3), a complex spectral signature is observed that stays relatively constant. When the scan reaches the nanolens again (spectra 4–6), a broad carbon signal is observed. The middle panel in Figure 4A gives another example of a complex spectral signature that is observed in two consecutive spectra (spectra 5, 6). Between these two measurement points, i.e., for several seconds, no new amorphous carbon species seems to be formed. The panel on the right in Figure 4A shows a broad background on which more-defined peaks appear over time. In the case of the panels on the left and right in Figure 4A, different carbonaceous species might have wandered into and out of the hot spot. Such thermally activated diffusion of individual molecules on the particle surface in conjunction with photo-induced electron transfer was proposed previously to be the cause of fluctuations in SERS spectra of amorphous carbon [32].

The sharp and intense peaks observed for some of the single silver nanolenses show a great variety and cannot exclusively be assigned to characteristic vibrations of DNA (Figure 4A). Strong fluctuations in the SERS signal of the molecule of interest can be characteristic for few-molecule SERS [3,33,34]. However, they more often originate from unwanted sample degradation to amorphous carbon [35,36,37,38]. When a sufficient number of spectra is averaged, few-molecule spectra from the analyte can be distinguished from amorphous carbon [4,8]. This should give a spectrum that resembles the ensemble spectrum of the respective analyte molecule. If signals originate from amorphous carbon instead, the averaging should yield the bands assigned to amorphous carbon. Applied to the data from twelve single silver nanolenses irradiated at 685 kW cm^−2^, we obtain such a broad amorphous carbon signal (Figure 4D, black). The broad double peak in the average is also obtained when those spectra that already have a strong background are left out and only those with distinctively sharp peaks are averaged (Figure 4D, grey). This provides further support that the spectral variation is caused by a diverse variety of individual carbon species, with the broad carbon signal as the sum spectrum. The very local probing of typical carbon vibrations by SERS was also discussed for other, low-dimensional nanomaterials, e.g., for single-walled carbon nanotubes [39].

In summary, we showed that on DNA-assembled plasmonic nanostructures, amorphous carbon is generated in situ, and that the broad background signal is constituted from individual spectra of this in situ-generated carbon with distinct peaks. The formation of amorphous carbon on single plasmonic nanostructures was followed over time, and both fluctuation and persistence of SERS signals from single or several different carbon species were observed. Especially for data from single plasmonic nanostructures providing very hot spots, these species provided spectra with sharp peaks, and special care has to be taken not to misinterpret them as the analyte signal. The experiments with dimer aggregates highlight the important influence of irradiation wavelength, power density and substrate. Silver dimers irradiated at 785 nm on silicon substrates showed an unusually high sensitivity towards amorphous carbon formation. For the excitation conditions used here, silver nanostructures are more likely to support the formation of amorphous carbon than gold nanostructures. This indicates that beyond factors related to plasmonics, the surface chemistry of the plasmonic substrate has to be recognized as an important influence. When aiming to avoid the formation of amorphous carbon, it proved more effective to decrease the power density than the integration time. Further strategies reported to prevent amorphous carbon formation comprise measurement at low temperature [1], in water [40], or under the exclusion of ambient oxygen and sulfur [8,25,41]. As an alternative to an inert gas atmosphere, the latter could be realized by a protective layer of graphene [42]. In DNA origami-based SERS, one of the molecular species closest to the metal surface is DNA. It was suggested that photocatalytic reactivity depends on the binding strength of the reacting molecules on the surface [29], which implies that the anchoring of the DNA strands on the particle surface can serve to tweak photo stability. Stronger anchoring, e.g., via multiple thiols, might provide more robust SERS systems. This and the aforementioned factors will have to be considered, should DNA origami-assembled plasmonic nanostructures be used for non-resonant single-molecule SERS or for other photocatalytic reactions than the generation of amorphous carbon. Plasmon-mediated chemical reactions for photocatalytic purposes have become the subject of intensive research [43,44]. DNA-assembled plasmonic substrates could develop into helpful tools to study such processes.

## 3. Materials and Methods

Raman measurements were carried out on a WITec alpha300 confocal Raman microscope (WITec GmbH, Ulm, Germany) with a 10× Nikon EPlan objective (numerical aperture 0.25) or a 100× Olympus MPlanFL N objective (numerical aperture 0.9), with a 50 µm pinhole and 600 gr/mm grating. The laser spot diameters estimated for the 10× objective are: 2.4 µm (λ = 488 nm), 2.6 µm (λ = 532 nm), 3.1 µm (λ = 633 nm), 3.8 µm (λ = 785 nm), for the 100× objective: 0.72 µm (λ = 532 nm). During a scan, the laser is continuously irradiating the sample, while the sample stage is moved in a continuous fashion along the scan path. The speed of this movement depends on the chosen integration time and step size. For the time series in the first part, areas of 170 × 190 µm^2^ were scanned with the 10× objective and a step size of 5 µm, such that the laser spots of two adjacent measurement points would not overlap. Each time series was conducted on a separate position of the sample. For the spectra from single silver nanolenses in the second part, areas of 25 × 25 µm^2^ were scanned with 0.5 µm steps, using the 100× objective. The number of scans before the amorphous carbon generation set on were observed to vary between different samples, but within a certain sample, the values were consistent. An assignment of background signals in the SERS spectra is given in the supporting information [45,46,47].

AFM measurements were carried out under dry conditions after the Raman measurements, with soft Tap150Al-G cantilevers in tapping mode to prevent sample manipulation.

Citrate-stabilized 60 nm gold nanoparticles from BBI Solutions (100 μL, 1 nM) were incubated with bis(p-sulfonatophenyl)phenylphosphine (BSPP) solution (20 μL, 2.5 mM) at 40 °C for 1 h. SDS (to a final concentration of 0.02%) and DNA coating strands (T_13_-SH, to a final concentration of 15 μM) were added. Samples were incubated at 40 °C for 30 min. The NaCl concentration was elevated in a stepwise manner, with at least 20 min of shaking at 40 °C between each step. First, 1 M NaCl was added in 20 mM steps, until a concentration of 100 mM was reached. Then, the NaCl concentration was increased to 150 mM, 200 mM, 300 mM, 400 mM, 600 mM and 750 mM, in the last four steps by adding 2.5 M NaCl.

Particles were purified by five cycles of centrifugation, supernatant removal and pellet resuspension in 400 μL 1× TAE, 11 mM MgCl_2_, 0.02% SDS.

Silver nanoparticles were coated with a similar protocol as described in ref. [16]. 60 nm silver nanoparticles were coated with dithiol-carrying DNA to counteract the weaker binding strength, but the dithiolated DNA still detached faster from the silver nanoparticles than monothiolated DNA from gold nanoparticles.

For the dimer assembly, triangular DNA origami scaffolds with four extended oligonucleotides on each face were assembled as described earlier [17]. The particles were bound by incubating 0.2 nM DNA origami solution with 0.4 nM DNA-coated 60 nm gold or silver nanoparticles for 3 h at room temperature. The products were purified by gel electrophoresis (1% agarose, 50 V for 90 min) and extracted by squeezing between two Parafilm-wrapped microscopy slides. The silver nanolenses were assembled as described elsewhere [16]. As in the reference, a streptavidin molecule was also bound on the DNA origami scaffold.

For deposition on silicon, substrates were plasma-cleaned, after which the nanostructure suspensions were added and incubated for 1 h with an excess of 10× TAE 120 mM MgCl_2_. The magnesium mediates binding between the negatively-charged DNA origami assemblies and the OH-groups on the silicon wafer. Substrates were washed with 4 mL EtOH/water 1:1 and blow-dried. For deposition on CaF_2_, samples were added to the substrate, incubated for 2 min, washed with 4 mL EtOH/water 1:1 and blow-dried.

FDTD simulations were carried out with Lumerical FDTD Solutions 8.6.3, for 60 nm dimers with 3 nm gap, 1.5 nm DNA coating around the particles, on a silicon substrate with a 2 nm silicon oxide layer, with exciting light polarized along the dimer axis and injected perpendicular to the dimer axis. Refractive indices: gold as determined by Johnson and Christy [48], silver, silicon and silicon dioxide as determined by Palik [49], DNA coating as determined by Thacker et al.: 1.7 [50], surrounding medium: 1.0. The simulation volume was surrounded by a perfectly matched layer absorbing boundary, the mesh size was 0.25 nm in the hot spot area.

## Supplementary Materials

Large-scale AFM images of gold and silver dimer samples, data from Figure 2 without offset, SERS spectra from gold and silver dimers under 785 nm excitation, SERS spectra from all sixteen single silver nanolenses and the corresponding AFM images, assignment of background signals in SERS measurements.
