# Supplementary material for: Amorphous Carbon Generation as a Photocatalytic Reaction on DNA-Assembled Gold and Silver Nanostructures
Source: Molecules. 2019 Jun 24;24(12):2324. doi: 10.3390/molecules24122324 (PMC6630242; doi:10.3390/molecules24122324)
Supplement: Supplementary file 1 [file molecules-24-02324-s001.pdf]

Amorphous carbon generation as photocatalytic reaction on DNA-assembled gold and silver nanostructures – Supporting Information

Christian Heck [1,2,3,#], Yuya Kanehira [1], Janina Kneipp [2,3] and Ilko Bald [1,2,*]

1 Institute of Chemistry, University of Potsdam, Karl-Liebknecht-Str. 24–25, 14476 Potsdam, Germany.
2 BAM Federal Institute for Materials Research and Testing, Richard-Willstätter-Str. 11, 12489 Berlin, Germany.
3 Department of Chemistry & SALSA, Humboldt Universität zu Berlin, Brook-Taylor-Str. 2, 12489 Berlin, Germany.
# Present address: School of Chemistry, Raymond and Beverly Sackler Faculty of Exact Sciences, Tel Aviv University, Tel Aviv, Israel.
* Correspondence: bald@uni-potsdam.de

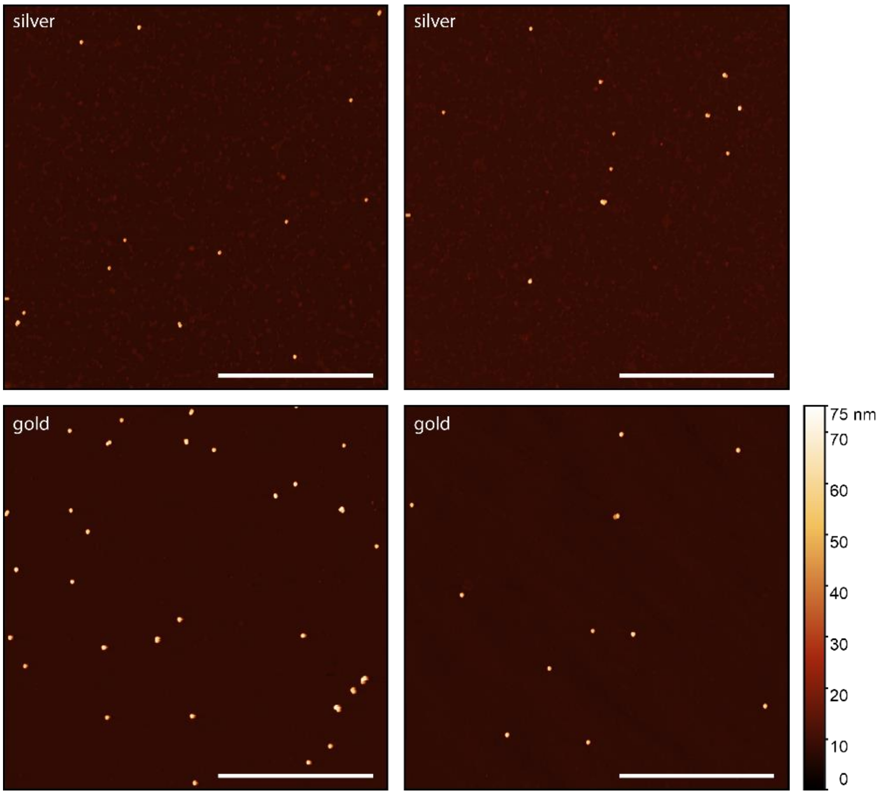

Figure S1. Representative AFM images of the 60 nm gold and silver dimer samples. Scale bars: 4 µm. The surfaces contain mostly monomers and dimers.

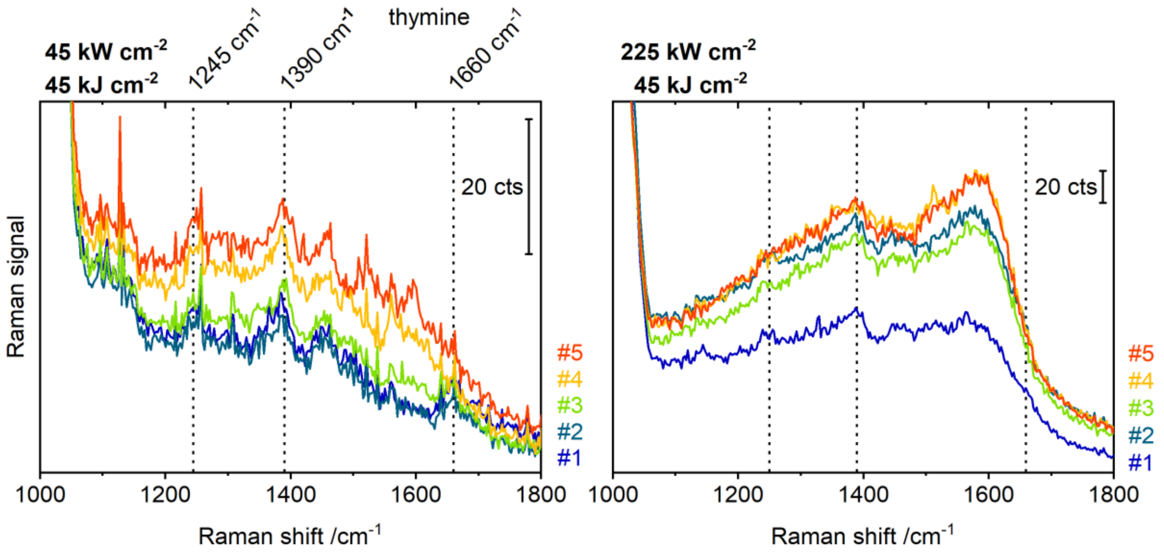

Figure S2. Average SERS signals of five consecutive scans over 60 nm silver dimers under 532 nm excitation, data from Figure 2, without offset. The series were carried out under different irradiation powers, but the integration time (left: 1 s, right: 0.2 s) was adjusted so as to achieve the same irradiation energy (45 kJ cm^{-2}). Characteristic thymine vibrations are marked by dotted lines.

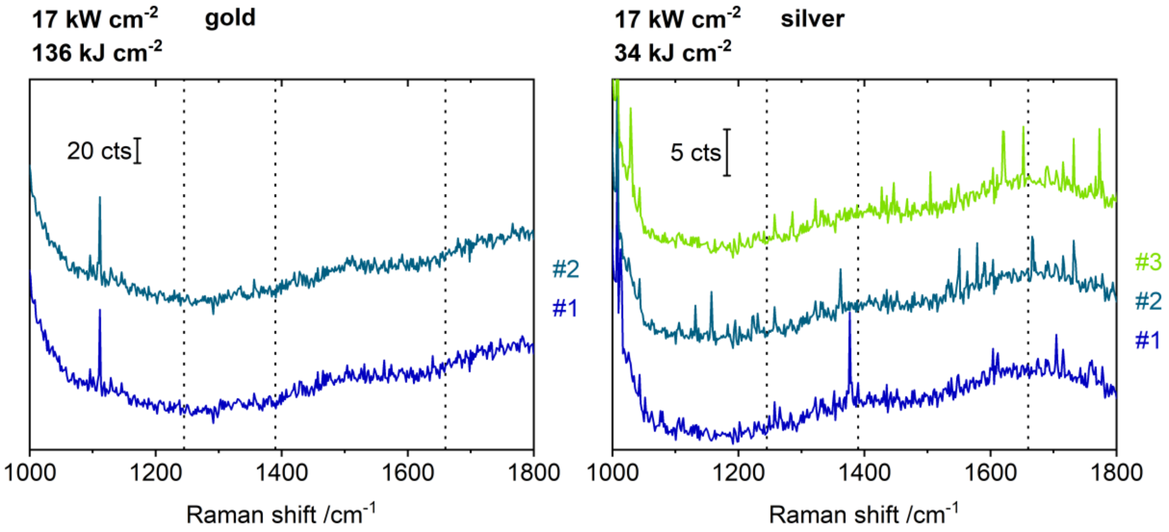

Figure S3. Average SERS signals of two consecutive scans over 60 nm gold (left) and silver (right) dimers on silicon substrate, under 785 nm excitation, with 8 s (left) or 2 s (right) integration per spot. Characteristic thymine vibrations are marked by dotted lines.

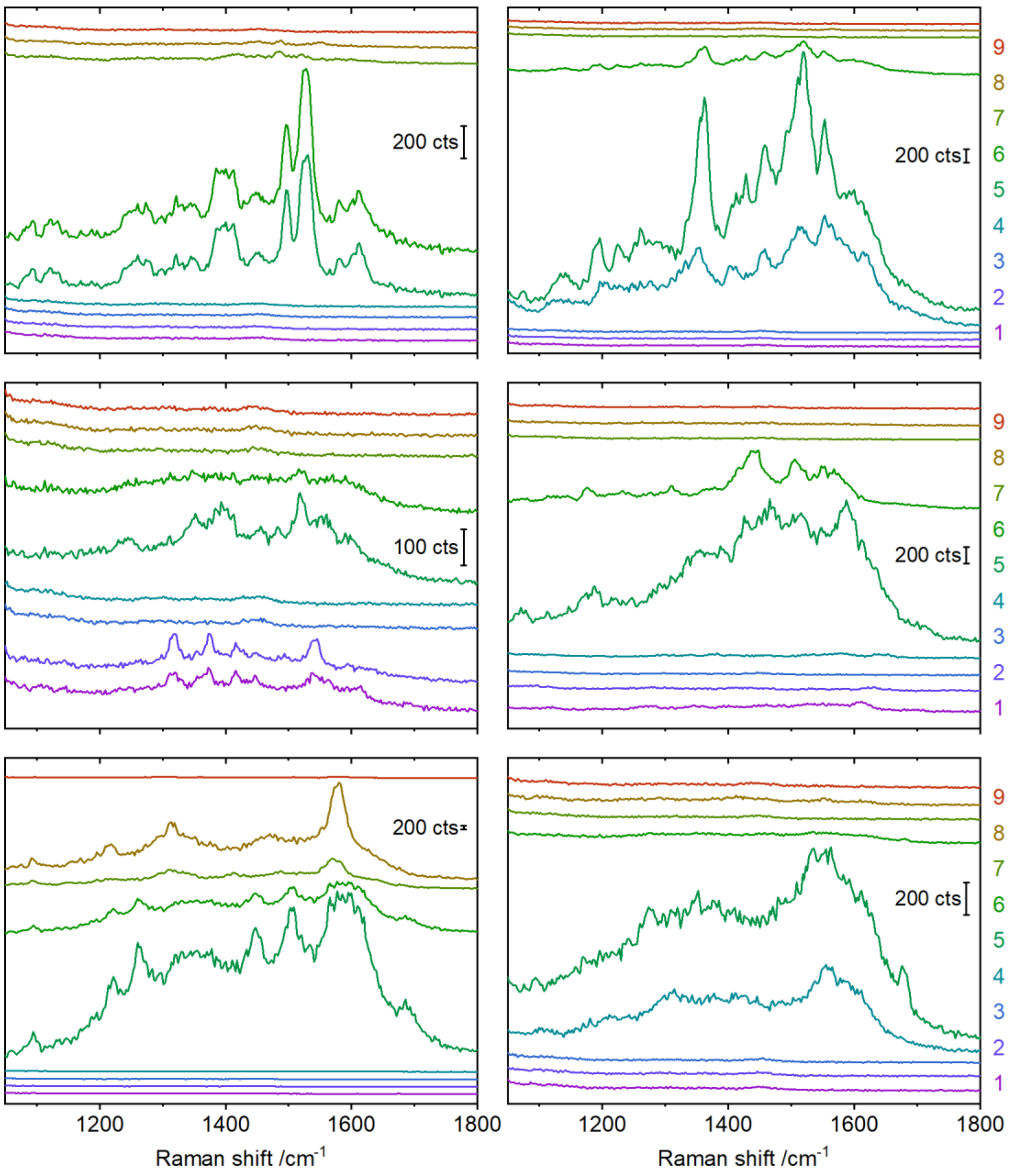

Figure S4. SERS spectra from scanning over single silver nanolenses (532 nm illumination, 4 s integration time per spot, 685 kW cm^{-2}), offset for clarity.

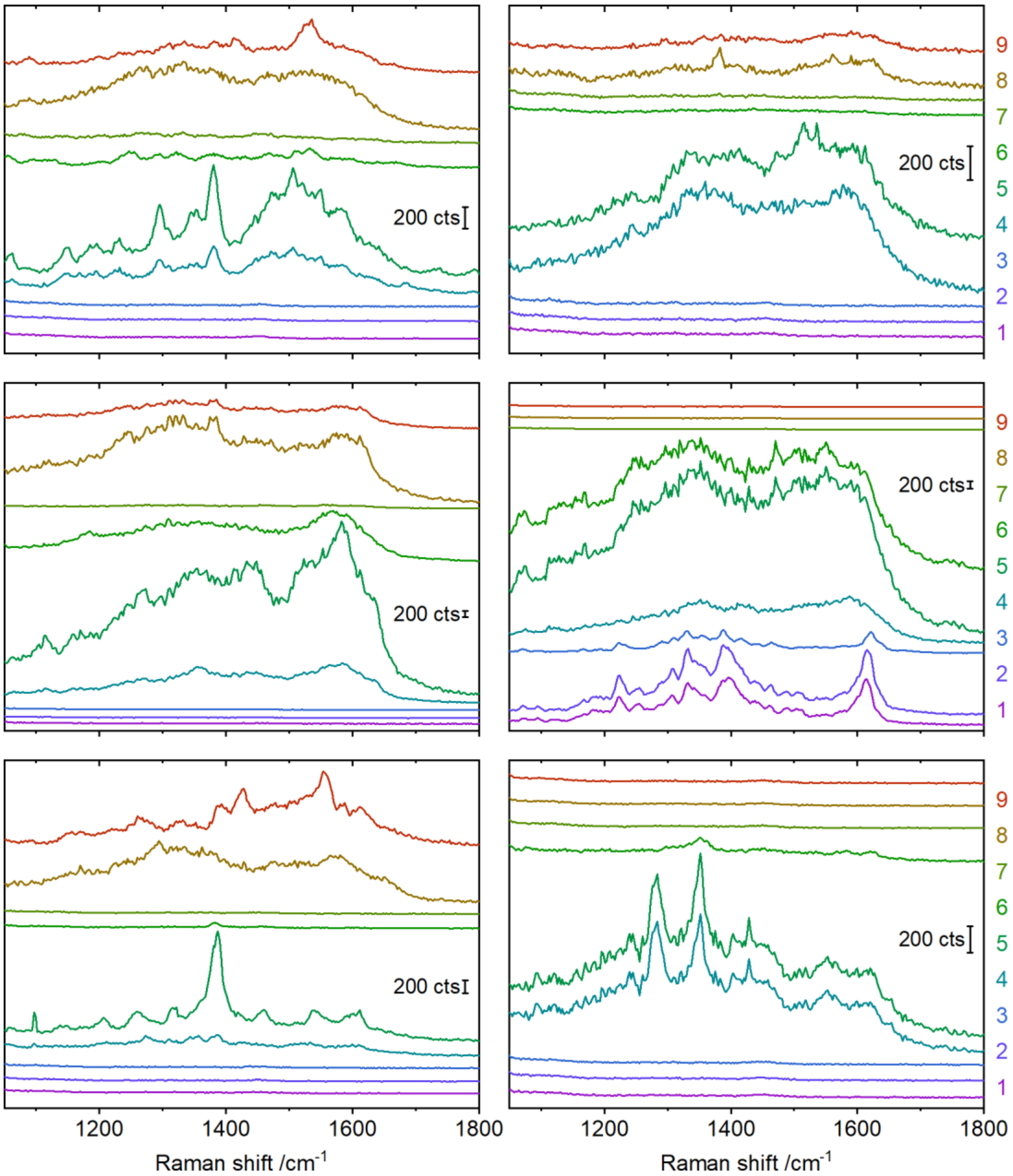

Figure S5. SERS spectra from scanning over single silver nanolenses (532 nm illumination, 4 s integration time per spot, 685 kW cm^{-2}), offset for clarity.

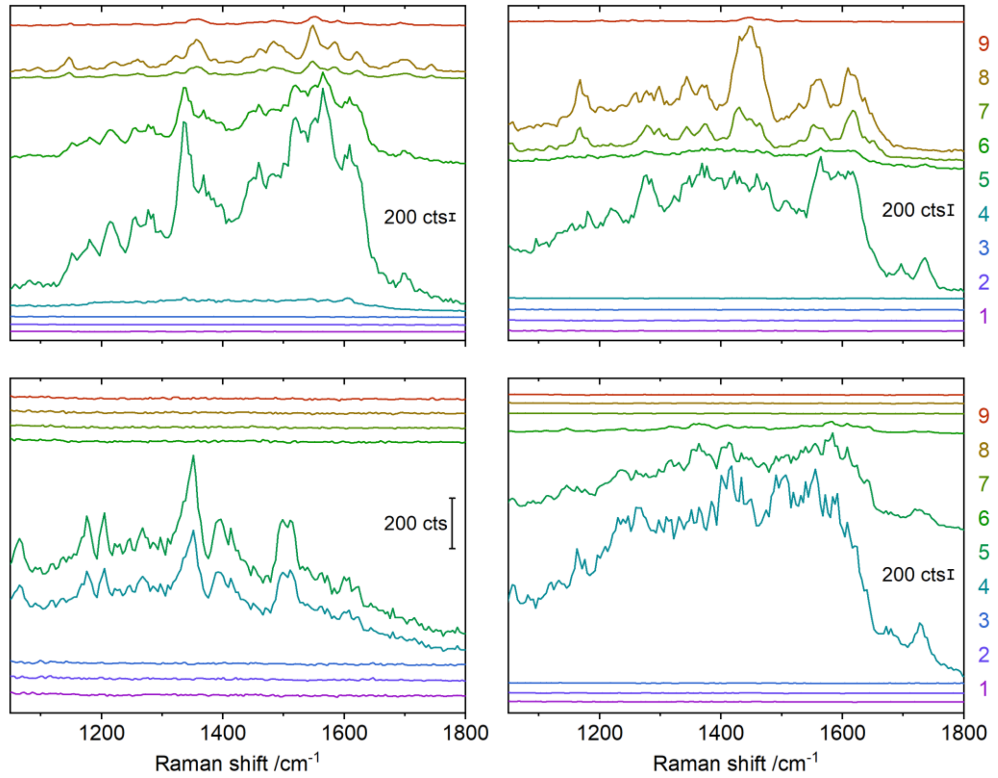

Figure S6. SERS spectra from scanning over single silver nanolenses (532 nm illumination, 4 s integration time per spot, 100 kW cm^{-2}), offset for clarity.

The spectra in Figures S4–S6 were obtained like those from Figure 4 in the main text. Raster scans over a silicon wafer with deposited silver nanolenses were conducted and since the step size in the scans was smaller than the laser spot size, each nanolens was sampled several times during a scan, creating a time series for each single silver nanolens. Figure 4C in the main text illustrates the scan path. Correlated AFM imaging confirmed that the structures were indeed single silver nanolenses. (See Figures S7–S9 for the corresponding AFM images.) SERS spectra from transient amorphous carbon spectra are known to exhibit a great variety of spectral signatures when -like here- individual hot spots are probed. [4] The reason for this lies in the multitude of different carbon species that are formed in the degradation process.

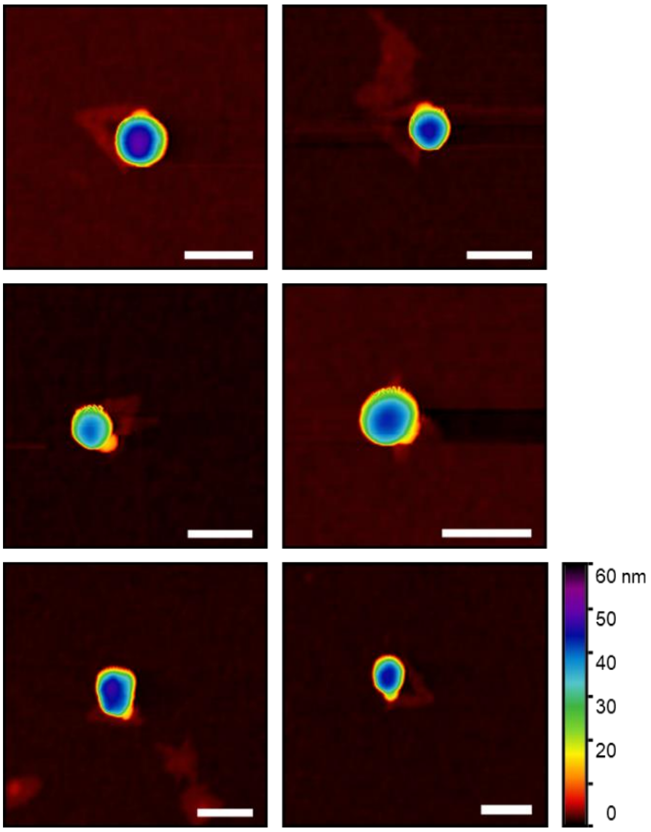

Figure S7. AFM images of the single silver nanolenses corresponding to the SERS spectra in Figure S4, ordered accordingly. Scale bars: 100 nm.

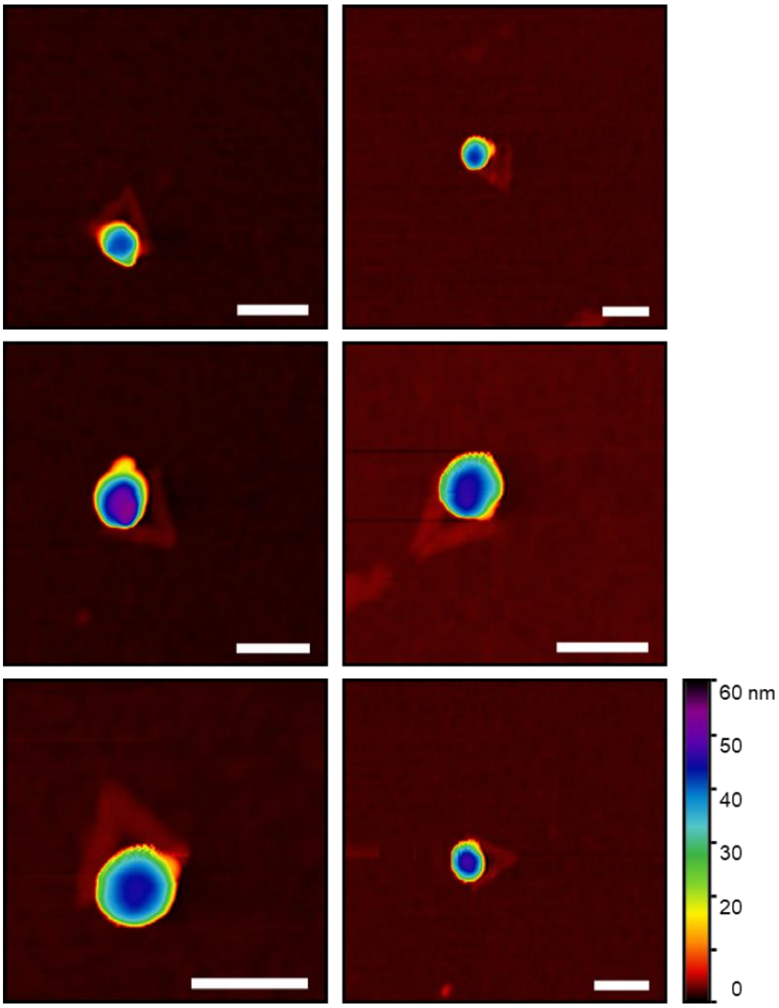

Figure S8. AFM images of the single silver nanolenses corresponding to the SERS spectra in Figure S5, ordered accordingly. Scale bars: 100 nm.

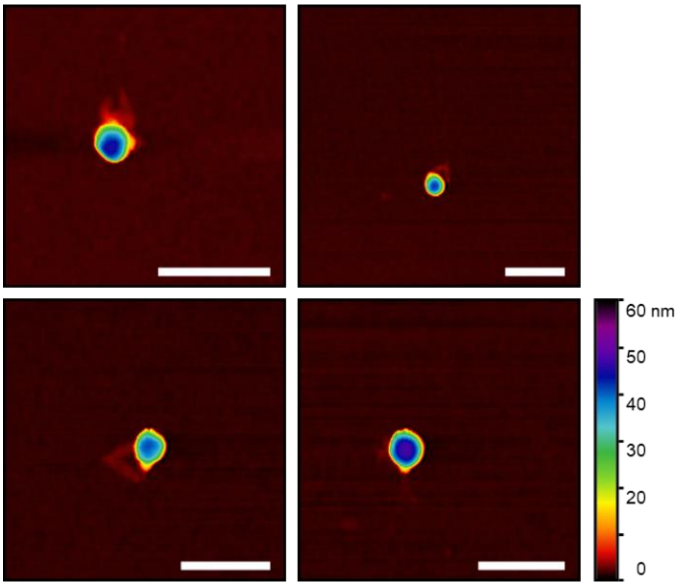

Figure S9. AFM images of the single silver nanolenses corresponding to the SERS spectra in Figure S6, ordered accordingly. Scale bars: 200 nm.

Assignment of background signals in SERS spectra:

900 cm^{-1}–1050 cm^{-1} 2nd order transverse optical phonon of silicon [45]

1070 cm^{-1} – 1160 cm^{-1} 2TO+TA (transverse optical + transverse acoustic) [45]

1240 cm^{-1} longitudinal optical phonon of SiO_2 [46]

1445 cm^{-1} 3rd order transverse optical phonon of silicon [45]

1556 cm^{-1} molecular oxygen [47]
